# Parent and Child Factors That Predict Who Helps Young Adult Children Pay for College

**DOI:** 10.1093/geroni/igaa057.1954

**Published:** 2020-12-16

**Authors:** Katrina Walsemann, Calley Fisk, Jennifer Ailshire

**Affiliations:** 1 University of South Carolina, Columbia, South Carolina, United States; 2 University of Southern California, Los Angeles, California, United States

## Abstract

In recent decades, the cost of higher education has exceeded the pace of inflation while wages have stagnated or declined. As such, young adult children may increasingly look to their parents and other family members, including grandparents, to help them pay for college. We use data from the National Longitudinal Survey of Youth 1979 to determine who financially contributes to a young adult child’s college education, restricting our sample to mid-life parents with at least one biological child who attended a 2-year or 4-year college and completed the college expenditures module in 2014 (n=3,525). For each college-going child, parents reported who paid for the student’s tuition – student, parents, grandparents, other family members, or a combination of these. Using multinomial logistic regression, we will estimate who paid for college as a function of parents’ social and economic characteristics when the child was 16 and the child’s gender and birth order.

